# Replicon RNA Viral Vectors as Vaccines

**DOI:** 10.3390/vaccines4040039

**Published:** 2016-11-07

**Authors:** Kenneth Lundstrom

**Affiliations:** PanTherapeutics, CH 1095 Lutry, Switzerland; lundstromkenneth@gmail.com; Tel.: +41-79-776-6351

**Keywords:** alphaviruses, flaviviruses, measles viruses, rhabdoviruses, antibodies, infectious diseases, tumors, disease protection

## Abstract

Single-stranded RNA viruses of both positive and negative polarity have been used as vectors for vaccine development. In this context, alphaviruses, flaviviruses, measles virus and rhabdoviruses have been engineered for expression of surface protein genes and antigens. Administration of replicon RNA vectors has resulted in strong immune responses and generation of neutralizing antibodies in various animal models. Immunization of mice, chicken, pigs and primates with virus-like particles, naked RNA or layered DNA/RNA plasmids has provided protection against challenges with lethal doses of infectious agents and administered tumor cells. Both prophylactic and therapeutic efficacy has been achieved in cancer immunotherapy. Moreover, recombinant particles and replicon RNAs have been encapsulated by liposomes to improve delivery and targeting. Replicon RNA vectors have also been subjected to clinical trials. Overall, immunization with self-replicating RNA viruses provides high transient expression levels of antigens resulting in generation of neutralizing antibody responses and protection against lethal challenges under safe conditions.

## 1. Introduction

Vaccine development against infectious diseases has classically been based on live attenuated or inactivated infectious agents [1]. Recently, the approach of vaccination with recombinantly expressed antigens and immunogens from viral and non-viral delivery systems has been introduced to the repertoire [2,3]. In this context, immunization with surface proteins and antigens has elicited strong humoral and cellular immune responses and vaccinated animals showed protection against challenges with lethal doses of infectious agents or tumor cells [4].

The types of non-viral vectors applied include liposomes [5], immunostimulatory complexes (ISCOMs) composed of adjuvant Quil A and peptides [6], and multiple antigen peptides (MAPs) also known as dendrimers [7]. A number of viral vectors based on adenoviruses, alphaviruses, avipoxiviruses, enteroviruses, flaviviruses, measles viruses (MV), rhabdoviruses, and vaccinia viruses have been engineered for vaccine development [3,8]. In this context, self-replicating RNA virus vectors have proven highly efficient for immunization studies in various animal models [9]. Among RNA viruses, rabies virus (RABV) and vesicular stomatitis virus (VSV) belonging to the rhabdovirus family carry a single-stranded RNA (ssRNA) genome of a negative polarity [10]. Likewise, MV possess a negative-sense ssRNA genome [11]. In contrast, flaviviruses and alphaviruses are of positive polarity. West Nile virus [12] and Kunjin virus [13] are the most common flaviviruses applied for immunization studies. Similarly, expression vectors have been engineered for alphaviruses such as Semliki Forest virus (SFV) [14], Sindbis virus (SIN) [15] and Venezuelan equine encephalitis virus (VEE) [16].

In this review, various self-replicating RNA virus vectors are described and their applications as recombinant virus particles, RNA replicons and layered DNA plasmids are compared. Moreover, examples are given of utilization of self-replicating RNA virus systems for immunization studies in various animal models to elicit humoral and cellular immune responses and to generate neutralizing antibodies, as well as protection against challenges with pathogens and tumor cells. Finally, a summary of clinical trials already conducted or in progress that apply self-replicating RNA viruses is presented. However, due to the large number of publications available, it is only possible to present key findings and examples of vaccine development for self-replicating viral vectors.

## 2. Self-Replicating RNA Expression Systems

Expression systems have been engineered for RNA viruses as described below. All ssRNA viruses share the feature of high level of RNA replication in the cytoplasm, which provides the basis for extreme transient expression of heterologous genes. However, the different polarities of the ssRNA genomes of self-replicating RNA viruses have required the design of vectors with specific features. Moreover, the viral vectors can be utilized in different forms as indicated for the individual types of viruses below.

### 2.1. Alphaviruses

**Figure 1:**
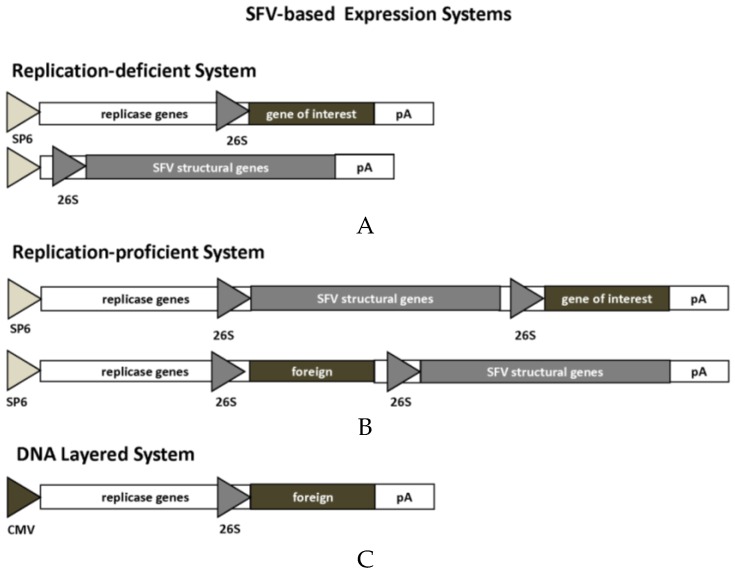
SFV-Based Expression Systems. (**A**) Replication-deficient system. In vitro transcribed RNA from expression and helper vectors are transfected into BHK-21 cells for generation of replication-deficient particles; (**B**) Replication-proficient system. In vitro transcribed RNA from full-length vector is transfected into BHK-21 cells for generation of replication-proficient particles; (**C**) DNA layered system. Plasmid DNA is transfected into host cells. 26S, SFV26S subgenomic promoter; CMV, cytomegalovirus promoter; pA, polyadenylation signal; SP6, SP6 phage RNA polymerase promoter.

Alphavirus-based expression systems have been developed in different formats for SFV [14], SIN [15] and VEE [16], here illustrated for SFV (Figure 1). In all cases, the basic component is represented by the alphavirus non-structural genes (nsP1-4), responsible for rapid and high quantity cytoplasmic RNA replication [17]. The replication-deficient system carries the gene of interest (GoI) downstream of the nsP1-4 genes in the alphavirus expression vector to be driven by the 26S subgenomic promoter. RNA replicon vectors can be generated by in vitro transcription for direct RNA immunization. In case of production of recombinant particles, in vitro transcribed RNA from an alphavirus helper vector is co-transfected or co-electroporated into baby hamster kidney (BHK) cells. The replication-proficient system utilizes a full-length vector, where the GoI can be introduced either downstream of the nsP1-4 genes or the structural genes. In vitro transcribed RNA can be applied for immunization, but due to the presence of full-length alphavirus genomic RNA, replication-proficient particles are generated. The DNA layered system applies plasmids carrying alternatively the nonstructural genes or the full-length genome and the GoI for direct immunization with DNA. All vector system approaches described above have proven efficient in immunization studies as presented below [4].

### 2.2. Flaviviruses

**Figure 2:**
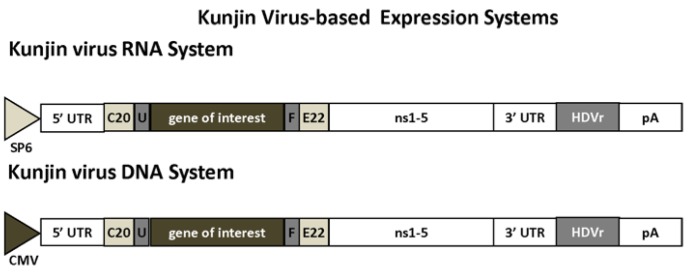
Kunjin Virus-Based Expression Systems. Expression vector based on Kunjin virus RNA and DNA expression systems. 3′ UTR, 3′ untranslated region; 5′ UTR, 5′ untranslated region; C20, first 20 amino acids of KUN C protein; CMV, cytomegalovirus promoter; E22, 22 last amino acids of KUN E protein; F, FMDV (foot-and-mouse disease virus) 2A autoprotease; HDVr, hepatitis delta virus ribozyme; ns1-5, nonstructural proteins; pA, polyadenylation signal; SP6, SP6 phage RNA polymerase promoter; U, mouse ubiquitin sequence.

Among flaviviruses, Kunjin virus [18], West Nile virus [19,20], yellow fever virus [21,22], dengue virus [23,24] and tick-borne encephalitis virus [25,26] have been engineered for the development of vectors for DNA, RNA and recombinant particle delivery. In Kunjin virus vectors, the GoI is inserted between the first 20 codons of the core protein (C20) and the last 22 codons of the envelope gene (E22) in frame with the rest of the viral polyprotein (Figure 2) [27]. The GoI is expressed initially as a fusion with the Kunjin virus polyprotein, which is then processed into individual proteins. Introduction of flanking FMDV-2A protease sequences allows the cleavage of Kunjin virus sequences from the expressed recombinant protein [28]. To facilitate vector production, a system has been engineered for transfection of Kunjin virus replicon RNA into the tetKUNCprMEC packaging cell line.

### 2.3. Measles Viruses

**Figure 3:**
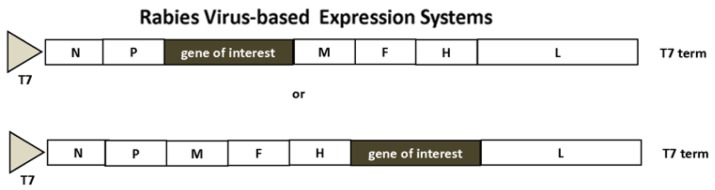
Measles Virus-Based Expression system. The measles virus structural proteins are flanked by T7 RNA polymerase promoter and the T7 RNA polymerase terminator. Foreign genes can be inserted between the P and M or H and L genes. H, MV hemagglutinin; L, MV L protein; M, MV matrix protein; N, MV nucleocapsid protein; T7, T7 RNA polymerase promoter; T7 term, T7 RNA polymerase terminator.

Expression systems have been engineered, whereby replicating MV is rescued from cloned DNA expression constructs [29] (Figure 3). Reverse genetics has allowed the rescue of recombinant measles virus in an HEK293 helper cell line, where foreign genes were introduced between the phosphoprotein (P) and the matrix protein (M) or between the hemagglutinin (H) and the large protein (L), respectively, in the measles virus genome [30]. Transfection of the helper cell line with recombinant MV constructs and a plasmid expressing the MV polymerase L gene is followed by transfer of syncytia to Vero cell cultures after 3 days, and recombinant MV particles are harvested when reaching 80%–90% cytopathic effects.

### 2.4. Rhabdoviruses

**Figure 4:**
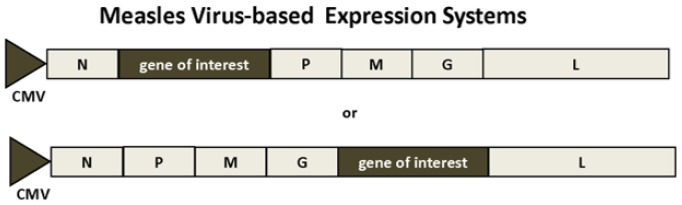
Rabies Virus-Based Expression Systems. The structural genes are from the HEP-Flury strain except the G protein from the CVS strain. Foreign genes can be inserted between the N and P or G and L genes, respectively. CMV, cytomegalovirus promoter; G, rabies G protein; L, rabies L protein; M, rabies matrix protein; N, rabies nucleocapsid protein.

Both RABV [10,31] and VSV [32,33] have been subjected to expression vector engineering (Figure 4). Similar to MV, reverse genetics has been applied for efficient recovery of VSV based on recombinant vaccinia virus, where the VSV N, P and L genes were inserted downstream of a T7 promoter and an internal ribosome entry site (IRES) [33]. The role of vaccinia virus has been to provide T7 RNA polymerase. However, vaccinia virus causes strong cytopathic effects in transfected cells, the vaccinia virus DNA polymerase contributes to homologous recombination between full-length genome and helper plasmids, and vaccinia virus may also contaminate recombinant virus stocks. For this reason, a BHK cell line stably expressing the T7 RNA polymerase was engineered as a vaccinia virus-free system for RABV [31]. In addition to rhabdovirus vectors, chimeric virus-like particles (VLPs) have been generated by expressing the VSV glycoprotein (VSV-G) in trans with the SFV replicon by introduction of a mutated SFV 26S promoter for packaging of infectious SFV pseudoparticles [34]. This system provides high biosafety standards as VSV-G shares no homology with the SFV genome.

## 3. Self-Replicating RNA Virus-Based Vaccines

Self-replicating RNA virus vectors have been frequently used for vaccine development against infectious diseases and various types of cancers [9]. Both vectors based on ssRNA viruses of positive (Kunjin virus, SFV, SIN, VEE) and negative (MV, RABV, VSV) polarity have been utilized for the expression of viral surface proteins and tumor antigens followed by immunization studies in animal models. Moreover, for vaccination, different approaches including recombinant particles, RNA replicons and layered DNA plasmids have been applied.

### 3.1. Vaccines against Infectious Diseases

The targets for vaccine development for infectious diseases comprise mainly surface antigens of pathogenic viruses (Table 1) and other infectious agents (Table 2). Obvious targets for vaccine development have been antigens of influenza virus and HIV. In this context, recombinant SFV particles expressing influenza nucleoprotein (NP) have demonstrated strong immune responses [35]. Similarly, VEE-based expression of hemagglutinin (HA) elicited strong immune responses and even provided protection against challenges with H5N1 virus in chicken [36]. Likewise, expression of the swine influenza virus HA H3N2 gene from VEE vectors protected swine from influenza virus challenges [37]. In another study, the swine influenza HA gene was expressed from replication-deficient alphavirus particles showing no spread of vaccine or reversion to virulence in the intended host (pig) or non-host (mouse) species [38]. Specific humoral and interferon-γ (IFN-γ) responses were observed in pigs, which were also protected against influenza virus challenges. Recombinant MV vectors carrying the HA gene have also been applied for vaccination studies [39]. Also VSV vectors have been utilized for vaccine development against influenza virus [40]. Instead of using full-length HA, expression of the stalk domain of HA generated chimeric HA (cHA) antigens. Both intramuscular and intranasal immunization of mice resulted in HA stalk-specific, cross-reactive antibodies. Prime-boost vaccination provided protection against lethal challenges with both homologous and heterologous influenza strains, which was significantly superior with intranasal administration.

For obvious reasons HIV has been a popular target for vaccine development. For instance, administration of Kunjin replicons expressing the HIV-1 gag antigen to BALB/c mice elicited gag-specific antibodies and protective gag-specific CD8+ T cell responses [41]. Interestingly, a single immunization with Kunjin virus particles induced 4.5-fold higher CD8+ T-cell responses and protection agains HIV challenges was obtained after two injections. Furthermore, RNA optimized Kunjin virus constructs for SIV Gag-Pol demonstrated improved effector memory and central memory responses as well as protection in primates [45]. Alphavirus vectors have also been employed for HIV vaccine development. Immunization with SFV particles expressing the Env [42] and gp41 [43] genes elicited humoral and cytotoxic T-lymphocyte (CTL) responses in mice. Interestingly, priming with a low dose (0.2 µg) DNA-based SFV replicon expressing the HIV Env and a Gag-Pol-Nef fusion prior to a heterologous boost with poxvirus (MVA) and/or HIV gp140 protein formulated in glycopyranosyl lipid A resulted in significantly enhanced immune responses [44]. Moreover, when macaques were immunized with a VSV vector carrying the SIV Env (smE660) gene neutralizing antibodies were obtained [46]. However, when challenged with SIVsmE660, all animals were infected. In contrast, vaccination with a combination of gag and Env resulted in immunity [47]. RABV vectors have also been employed for the expression of SIV Env and gag in macaques [48]. Although immune responses were detected for RABV glycoprotein G, no cellular responses were obtained against SIV antigens. However, replacing the RABV G with VSV G resulted in SIV-specific immune responses and immunized macaques were protected against SIV challenges.

A number of immunization studies have targeted such lethal viruses as Ebola and Lassa viruses. For instance, dose-dependent protection against Ebola virus was achieved in guinea pigs when immunized with Kunjin virus particles expressing the Ebola virus wild-type glycoprotein GP or a mutant GP (D637L) [49]. Similarly, African green monkeys were subcutaneously immunized with Kunjin particles carrying the Ebola GP D673L mutant [50]. Protection of three out of four primates was obtained against challenges with Zaire Ebola virus. Application of VSV vectors expressing the Ebola GP gene has also provided protection of macaques after challenges with the West African EBOV-Makuna strain [51]. Likewise, protection against three different Ebola strains was achieved by expression of Ebola GP from VSV vectors [52]. Alphavirus vectors have also been utilized for vaccine development against Ebola virus. In this context, RNA replicons derived from an attenuated VEE strain were applied for the expression of Ebola GP and nucleoprotein (NP) [53]. Immunization studies showed that VEE-GP alone or in combination with VEE-NP provided protection of both BALB/c mice and guinea pigs. In contrast, VEE-NP alone did not confer protection in guinea pigs, but did in mice. In another study, C57BL/6 mice were immunized with VEE particles expressing Ebola NP, which protected animals from Ebola virus challenges [54].

VSV vectors have been subjected to immunization studies for expression of the Lassa virus glycoprotein (strain Josiah, Sierra Leone), which generated protection in guinea pigs after a single prophylactic injection [55]. It was also shown that macaques were protected against challenges with the genetically distinct Liberian Lassa virus isolate. Importantly, previous VSV-based Lassa virus vaccination did not have an impact on immunization with VSV-Ebola GP particles [77]. Furthermore, alphaviruses have been used for vaccine development against Lassa virus [56]. Guinea pigs immunized with VEE particles expressing Lassa virus glycoprotein or nucleoprotein showed protection against lethal challenges with Lassa virus. Furthermore, a dual expression approach for Ebola and Lassa virus glycoproteins was engineered, which led to protection against both Ebola and Lassa virus challenges.

A number of other viral antigens have been subjected to vaccine development. Currently, relevant targets comprise dengue virus, severe acute respiratory syndrome coronavirus (SARS-CoV) and Middle East respiratory syndrome corona virus (MERS-CoV). For instance, mice immunized with VEE particles expressing the SARS-CoV glycoprotein provided protection against lethal SARS-CoV challenges [57]. Furthermore, mice were immunized with MV vectors expressing the MERS-CoV glycoprotein, which resulted in induction of T-cell and antibody responses and protection against lethal doses of MERS-CoV [58]. Respiratory syncytial virus (RSV) has also been targeted with recombinant MV vectors by expression of the RSV fusion protein (RSV-F) [39]. Immunization of cotton rats induced neutralizing antibodies against RSV and protected against RSV infection in the lungs. In another application, lipid nanoparticle (LNP) formulations were engineered for VEE RNA replicons, which demonstrated protection against RSV challenges in vaccinated mice [59]. Furthermore, immunization of African green monkeys with VEE particles expressing human RSV-F and metapneumovirus F (hMPV-F) proteins generated RSV-F and MPV-F-specific antibodies resulting in protection against RSV and MPV challenges [60]. In the context of dengue virus vaccines, a hybrid MV vector expressing the hepatitis B surface antigen (HBsAg) and the dengue virus 2 envelope protein (DV2) elicited neutralizing antibodies against MV, HBsAg and DV2 [61]. In another study, MV-DV2 vaccination of mice generated IFN-γ and DV2 antibody responses and protection against four DV serotypes [62]. Alphavirus vectors have also been evaluated for dengue vaccine development. Expression of two configurations of dengue virus E antigen (prME and E85) provided protection in macaques [63]. Moreover, a single immunization of BALB/c mice was sufficient to induce neutralizing antibodies and T-cell responses [64]. The neonatal immunization was durable, could be boosted later in life and provided protection against challenges with dengue virus. Additional viral targets evaluated for vaccine development are listed in Table 1.

Vaccine development has also been extended to other infectious diseases than caused by viral infections (Table 2). In this context, mice vaccinated with SFV vectors expressing the *Plasmodium falciparum* Pf332 antigen elicited immunological memory [68]. Similarly, strong immunity and long-term protection against *Mycobacterium tuberculosis* was obtained in mice immunized with SIN plasmid DNA vectors carrying the *M. tuberculosis* 85A antigen (Ag85A) [69]. Furthermore, expression of the botulinum neurotoxin A from layered SFV DNA plasmids elicited antibody and lymphoproliferative responses in immunized BALB/c mice [70]. Co-expression of granulocyte-macrophage colony-stimulating factor (GM-CSF) enhanced the immune response. Replication-deficient SFV particles carrying the *Brucella abortus* translation initiation factor 3 (IF3) were subjected to immunization studies in BALB/c mice, which resulted in protection against challenges with the virulent *B. abortus* strain 2308 [71]. In another study, SIN vectors were utilized for the expression of the protective antigen (PA) for *Bacillus antracis* in Swiss Webster mice leading to the generation of specific and neutralizing antibodies and partial protection against challenges with pathogenic bacteria [72].

Recombinant SIN vectors were applied for the expression of a class I major histocompatibility complex-restricted 9-mer epitope of the *Plasmodium yoelii* circumsporozoite protein (CS), which induced a strong epitope-specific CD8+ T-cell response and a high degree of protection against malaria infection in mice [73]. Another approach to develop malaria vaccines involves the application of a live attenuated MV vaccine expressing recombinant antigens against malaria [78]. A modified replication-competent VSV vector pseudotyped with the glycoprotein of the lymphocytic choriomeningitis virus (VSV-GP) expressing ovalbumin (OVA) induced humoral and cellular immune responses after a single administration in mice [74]. Due to the generation of neutralizing antibodies against VSV, immunization boosters were only possible for VSV-GP-OVA. CTL responses of similar potency as obtained for state-of-the-art adenovirus administration were observed and complete protection against challenges with *Listeria* monocytogenes was obtained in mice. In the context of prion disease, SFV DNA, RNA and recombinant particles were employed for the expression of prion protein (PRNP), which allowed generation of monoclonal antibodies against PRNP in immunized mice [75]. Although not directly applied for vaccine development, the generated monoclonal antibodies will be useful for basic research and diagnostics for prions. Alphavirus vectors have also been applied for the development of vaccines against Staphylococcus enterotoxin B (SEB) [76]. Subcutaneous administration of VEE particles expressing SEB resulted in protection against challenge of wild-type SEB in mice.

### 3.2. Vaccines against Cancer

A number of immunization studies have been carried out with self-replicating RNA virus vectors in the area of oncology (Table 3). For instance, attenuated oncolytic MV strains such as the Edmonston-B (MV-Edm) strain demonstrated anti-tumor activity [79]. The MV-Edm strain does not cause any significant cytopathic effect in normal tissue, but can selectively infect and replicate in tumor cells based on evaluations in cell lines, primary cancer cells and xenograft and syngeneic models for B-cell Non-Hodgkin lymphoma [80], ovarian cancer [81], glioblastoma multiforme [82], breast [83] and prostate [79] cancers. In this context, tumor regression was obtained in SCID mice with human lymphoma xenografts after intratumoral injection of MV-Edm [80]. Moreover, co-administration of MV vectors expressing carcinoembryonic antigen (CEA) and thyroidal sodium iodide symporter (NIS) in mice with SKOV3ip.1 ovarian xenografts showed superior tumor regression in comparison to treatment with either MV-CEA or MV-NIS alone [81]. To improve delivery and enhance efficacy, CD46 and signaling lymphocytic activation molecule (SLAM) ablating mutations in the hemagglutinin protein in combination with the display of a single-chain antibody against the epidermal growth factor receptor (EGFR) were incorporated into MV vectors for tumor targeting [82]. Tumor regression and significantly extended survival were observed after intratumoral administration of MV. Evaluation of MV-CEA delivery in an MDA-MB-231 mammary tumor model revealed a significant delay in tumor growth and prolonged survival [83]. Moreover, intratumoral administration of MV-CEA vectors showed tumor growth delay and improved survival in a subcutaneous PC-3 xenograft model [79].

Rhabdoviruses have also been applied in cancer therapy [84]. VSV vectors lack pre-existing immunity in humans and have demonstrated high susceptibility of cancer cells. Particularly, VSV vectors have been subjected to aggressive pancreatic ductal adenocarcinoma (PDAC) showing superiority to Sendai virus and RSV in 13 clinically relevant human pancreatic cell lines, although the response varied from one cell line to another [85]. Moreover, evaluation in ten PDAC cell lines of three VSV vectors expressing the wild-type matrix protein or ∆M51 showed activation of VSV-mediated apoptosis [86]. However, high constitutive expression of IFN-stimulated genes (ISGs) was discovered in three cell lines, which also contributed to resistance to apoptosis.

Kunjin virus replicons expressing the granulocyte colony-stimulating factor (GM-CSF) have been subjected to intratumoral administration, which resulted in cure in less than 50% of mice with established CT26 colon carcinoma and B16-OVA melanomas [87]. Subcutaneous injection led to regression in CT26 lung metastasis. Moreover, Kunjin vectors were engineered to express a CTL epitope of HPV16 E7 protein, which induced E7-directed T-cell responses and provided protection against challenges with an E7-expressing epithelial tumor in mice [88]. In this study, the Kunjin VLPs were more effective than RNA replicons or DNA vectors.

Alphavirus vectors have been applied in many studies on cancer vaccines [4,8]. In principle, tumor-associated antigens (TAAs), immunomodulating cytokines and combination therapies of TAAs and cytokines, TAAs and antibodies, cytokines and antibodies and even microRNAs (miRNAs) have been evaluated. In this context, intratumoral injection of SFV particles expressing enhanced green fluorescent protein (EGFP) showed apoptosis induction in mice implanted with human non-small cell lung carcinoma H353a cells in mice [109]. Furthermore, intratumoral administration of SFV particles into BALB/c mice with implanted sarcoma K-BALB sarcoma and CT26 colon tumors resulted in significant tumor growth inhibition [103]. Also, vaccination of mice with SFV RNA replicons expressing β-galactosidase showed protection against challenges with colon tumor cells [100]. Only a single intratumoral injection of 1 μg of SFV-LacZ RNA resulted in 10–20 days of survival extension in mice with existing tumors. Similarly, SIN-LacZ vectors demonstrated therapeutic efficacy in a mouse CT26 colon carcinoma model [101]. Despite not targeting specifically CT26 cells, SIN vectors showed susceptibility to mediastinal lymph nodes (MLNs), which induced effector and memory CD8+ T-cells displaying robust cytotoxicity. The well-characterized human carcinoembryonic antigen (CEA) elicited neutralizing antibodies after VEE VLP immunization [120]. Moreover, melanoma antigens such as tyrosine-related proteins TRP-1 and TRP-2, gp100 and melanoma antigen tyrosinase (Tyr) have been expressed from VEE vectors [94,112]. For instance, immunization of mice with VEE-TRP-2 particles resulted in growth inhibition of B16 transplantable melanoma and strong therapeutic potency [111]. Vaccination with VEE-TRP-2 VLPs was more efficient than the combination of VEE-gp100 and VEE-Tyr particles. Furthermore, VEE particles carrying the Tyr gene induced immune responses and tumor protection in mice after administration of VEE VLPs alone or an initial vaccination with plasmid DNA followed by boosting with VEE VLPs [112].

Breast cancer has been targeted in several therapeutic and prophylactic vaccine studies. SIN plasmid DNA carrying the neu gene were subjected to immunization of mice resulting in inhibition of growth of challenged A2L2 tumor cells [94]. Interestingly, vaccination two days after tumor challenge was inefficient. In contrast, immunization in a prime-boost protocol with SIN-neu DNA followed by adenovirus vectors carrying the neu gene prolonged the survival of mice. Due to their potency of stimulation of antigen-specific T-cells, dendritic cells (DCs) were transduced by VEE-neu particles, which resulted in high-level transgene expression, DC maturation and secretion of pro-inflammatory cytokines [95]. Robust neu-specific CD8+ T-cell and anti-neu IgG responses were observed after a single immunization. Moreover, regression of large established tumors was obtained. Another TAA attractive for immunotherapy is the six-transmembrane epithelial antigen of the prostate (STEAP), which has demonstrated up-regulation in multiple cancer cell lines [121]. Transgenic adenocarcinoma of mouse prostate (TRAMPC-2) tumor-bearing mice pre-immunized with VEE VLPs expressing STEAP demonstrated a strong immune response and a significantly prolonged overall survival [116]. The therapeutic affect was assessed for mice with 31-day-old tumors, which resulted in a modest but significant delay in tumor growth. Furthermore, VEE VLPs have been applied for expression of the prostate stem cell antigen (PSCA) in TRAMP mice, where the initial immunization with a PSCA DNA plasmid was followed by VEE-PSCA VLP delivery [117]. The outcome was a specific immune response and protection against tumor challenges in 90% of TRAMP mice. Also, the prostate-specific membrane antigen (PSMA) has been expressed from VEE vectors demonstrating strong humoral and cellular immune responses in subcutaneously immunized mice [115]. VEE VLPs expressing the prostate-specific antigen (PSA) were used for immunization of mice followed by a challenge with TRAMP cells [118]. The VEE VLPs were capable of infecting DCs in vitro and induced a robust PSA-specific response in vivo. Tumors in vaccinated animals showed low PSA expression levels and tumor growth was significantly delayed.

The P815A antigen is expressed in P815 mastocytoma tumors, which triggered an immunization study on the P1A gene coding for the PP815A antigen [119]. SFV particles expressing the P1A gene elicited strong CTL responses and protected immunized mice from challenges with P815 tumors. Other interesting TAA vaccine targets have been the E6 and E7 proteins of the human papilloma virus (HPV). Immunization with SFV particles expressing HPV type 16 E6,7 showed strong HPV-specific CTL activity and eradicated HPV-transformed tumors [97]. Similarly, immunization of mice with VEE particles carrying the HPV16 E7 gene prevented tumor development and eliminated established tumors in 67% of vaccinated animals [99]. In another study, tattoo injection [122] of SFV-HPV E6,7 particles resulted in antigen expression in both the skin and draining lymph nodes leading to ten-fold lower antigen levels in comparison to intramuscular administration [98]. However, tattoo injection provided higher or equal levels of immune responses.

Cytokines have played an important role in immunotherapy and vaccine development [8]. For instance, interleukin-12 (IL-12) has been expressed from both SFV and SIN vectors. In this context, SFV vectors expressing IL-12 induced tumor regression with long-term tumor-free survival in the MC38 colon carcinoma model [102]. Repeated intratumoral administration increased the anti-tumor response. In another study, immunization with SFV-luciferase and SFV-IL-12 particles was evaluated in a woodchuck model in which hepatocellular carcinoma (HCC) is induced by the infection by woodchuck hepatitis virus (WHV) [105]. High luciferase expression levels were observed in tumors and IL-12 secretion was measured in the serum after intratumoral injections. In tumor-bearing woodchucks, partial tumor remission was seen. Tumor volumes were reduced by 80%, but tumor growth was restored with time. The plasmid vector pTonL2(T)-mIL12, which provides liver-specific and inducible IL-12 expression, has been compared to SFV-IL-12 particle delivery in a L-PK/c-myc transgenic mouse model of HCC [106]. Overexpression of the c-myc gene in the liver of the transgenic animals induces spontaneous hepatic tumors with characteristics similar to human HCCs. Intratumoral administration of SFV-IL-12 resulted in tumor growth arrest and 100% survival rates. Mice treated with plasmid DNA showed a slightly lower survival rate despite higher IL-12 and IFN-γ levels in serum. The strong anti-tumor response in SFV-IL-12-treated mice was most likely due to the apoptosis and type 1 IFN response induced by SFV particles. Recombinant SIN particles were demonstrated to target tumor cells in SCID mice, which encouraged intraperitoneal injection of SIN-IL-12 particles in mice with established ovarian tumors [113]. The treatment resulted in systemic targeting and eradication of tumor cells without any adverse effects observed. Glioma-bearing mice were immunized with SFV-IL-12 particles, which induced apoptosis of glioma cells and facilitated the uptake of apoptotic cells by DCs and provided prolonged survival of vaccinated animals [91]. Moreover, DCs isolated from bone marrow were transduced with SFV vectors expressing IL-12 for the treatment of brain tumor-bearing mice [92]. The outcome was prolonged survival of immunized animals. In another study, SFV-IL-12 particles were tested in rat RG2 gliomas [93]. Low dose (5 × 10^7^ VLPs) treatment resulted in a 70% reduction in tumor volume, whereas high-dose (5 × 10^8^ VLPs) showed an 87% reduction in tumor volume. Moreover, intratumoral administration of 10^6^ oncolytic SFV particles expressing EGFP generated significant tumor regression in melanoma-bearing SCID mice [123]. Other cytokines such as Il-18 have also been evaluated for alphavirus-based expression in ovarian and colon cancer models [104]. The enhanced SFV10E vector, which provides ten-fold higher levels of expression than the conventional SFV vector [124], was applied for immunization of BALB/c mice [114]. After in vitro verification of secretion of active IL-18, mice with subcutaneous K-BALB and CT26 tumors were injected with SFV-IL-18 particles, which led to tumor regression and disappearance of tumors in some treated animals. Moreover, GM-CSF, an immunostimulatory cytokine, has been expressed from SFV vectors [114]. Intraperitoneal administration of SFV-GM-CSF particles was evaluated in an ovarian mouse tumor model, which resulted in activation of macrophages to tumor cytotoxicity. Although no prolongation in survival of tumor-bearing mice was achieved, tumor growth was inhibited for two weeks.

Among the growth factors targeted for vaccine development, the vascular endothelial growth factor receptor 2 (VEGFR-2) was introduced into the SFV vector [96]. Immunization of mice with SFV-VEGFR-2 particles resulted in substantial inhibition of both tumor growth and spread of pulmonary metastases. Furthermore, vaccination led to tumor inhibition in mice with established CT26 colon tumors and metastatic 4T1 mammary tumors. In another approach, SFV particles carrying the endostatin gene were administered to mice bearing B16 brain tumors [89]. The treatment resulted in a substantial reduction in intratumoral vascularization in tumor sections and a significant inhibition of tumor growth. Endostatin serum levels were three-fold higher 7 days after intravenous administration of SFV-endostatin in comparison to administration of the retrovirus-based GCsap-Endostatin promoting inhibition of angiogenesis in established tumors. In another approach, SIN vectors have been employed for the expression of a fusion protein of HPV16 E7 protein and calreticulin (CRT), an ER Ca2^+^-binding transporter participating in antigen processing and presentation with major histocompatibility complex (MHC) class I [108]. Immunization of mice bearing E7-expressing tumors with SIN-E7-CRT particles significantly increased E7-specific CD8+ T-cell precursors and a strong anti-tumor response. Furthermore, a significant reduction in lung tumor nodules was observed in immuno-compromised BALB/c mice.

Combination therapy has been evaluated for alphavirus-based gene delivery. For instance, SFV layered DNA vectors were engineered to express one to four domains of VEGFR-2 and IL-12 [110]. Co-immunization with SFV replicon DNA expressing survivin and β-hCG antigens was verified in mice resulting in efficient humoral and cellular immune responses against survivin, β-hCG and VEGFR-2. Moreover, tumor growth was inhibited and the survival rate in a B16 melanoma mouse model was improved. Furthermore, immunization with SFV HPV E6/E7 was combined with sunitib and a single low-dose of irradiation, which enhanced the intratumoral ratio of anti-tumor effector cells to myeloid-derived suppressor cells [107]. Triple treatment of tumor-bearing mice demonstrated enhanced anti-tumor efficacy and provided 100% tumor-free survival.

An interesting approach comprises the introduction of micro RNA-124 (miR-124) into an SFV4 vector [90]. As IFN-1 tolerance has been associated with the SFV nsP3-nsP4 genes, conditionally replicating SFV4-miR-124 virus was able to replicate in neurons and allowed targeting of gliomas otherwise sensitive to IFN-1. Evaluation of CT-2A mouse astrocytoma cells and IFN-1 pretreated human glioblastoma cells showed increased oncolytic potency. Moreover, a single intraperitoneal injection of SFV4-miR-124 into mice with implanted CT-2A orthotopic gliomas showed significant inhibition of tumor growth and improved survival rates.

### 3.3. Clinical Trials

Self-replicating RNA virus vectors have been subjected to several clinical studies, albeit at an inferior level in comparison to adenovirus, AAV and lentivirus vectors. For instance, healthy volunteers were subjected to low-dose (3 × 10^5^ pfu) immunization with the VSV-based Ebola vaccine (rVSV-ZEBOV) expressing the Zaire Ebola virus glycoprotein in a double-blinded study in comparison to a previous study with a high dose (5 × 10^7^ pfu) [125]. No serious adverse events occurred and the overall safety was good. The low-dose immunization improved early tolerability, but generated inferior antibody responses and failed to prevent vaccine-induced arthritis, dermatitis or vasculitis. Furthermore, VSV particles expressing the HIV-1 gag gene were evaluated in a clinical trial on safety and immunogenicity [126]. In the randomized double-blinded placebo-controlled dose-escalation study, healthy HIV-negative volunteers received 4.6 × 10^3^ to 3.4 × 10^7^ pfu of rVSV HIV-1 gag vaccine intramuscularly at months 0 and 2. All vaccinated individuals showed antibody responses against VSV, and gag-specific T-cell responses were detected in 63%. Overall, the safety profile was good.

Alphaviruses have been subjected to some gene therapy and vaccine studies. In one approach, replication-deficient SFV particles were encapsulated in liposomes to promote passive targeting of tumors [127]. Initially, intraperitoneal administration of encapsulated SFV-LacZ particles showed enhanced accumulation of β-galactosidase in SCID mice implanted with LNCaP prostate tumors. Liposome-encapsulated SFV particles expressing the p40 and p35 subunits of IL-12 generated active secreted IL-12 in BHK-21 cells [128]. Next, encapsulated SFV-IL-12 particles were administered intravenously in terminally ill melanoma and kidney carcinoma patients in a phase I clinical trial. The patients showed a five to ten-fold increase in IL-12 plasma levels. The maximum tolerated dose was determined to 3 × 10^9^ infectious particles and the safety profile was good. A phase I dose-escalation trial was conducted in prostate cancer patients with VEE particles expressing PSMA [129]. Patients with castration-resistant metastatic prostate cancer (CRPC) received up to five doses of either 0.9 × 10^7^ IU or 0.36 × 10^8^ IU of VEE-PSMA particles at weeks 1, 4, 7, 10 and 18. The study showed no toxicity and good toleration of the vaccination. However, only weak PSMA-specific immune responses were detected and no clinical benefits obtained. In another clinical trial, VEE particles expressing the CEA tumor antigen were demonstrated to efficiently infect DCs [120]. The VEE particles could be repeatedly administered and overcame high titers of neutralizing antibodies and elevated regulatory T cells (Tregs), which allowed induction of clinically relevant CEA-specific T cell and antibody responses. In another approach, VEE particles expressing the cytomegalovirus (CMV) gB and pp65/IE1 fusion protein were evaluated in a phase I randomized, double-blinded clinical trial [67]. Intramuscular or subcutaneous immunization at weeks 0, 8 and 24 of CMV seronegative adult volunteers showed good tolerance with only mild to moderate local reactions and no clinically important changes. Neutralizing and multifunctional T-cell responses against CMV antigens were detected in all vaccinated individuals.

## 4. Conclusions

Self-replicating RNA viruses represented by ssRNA viruses of both negative and positive polarity have been subjected to engineering of efficient gene delivery vectors, which can be applied in the form of recombinant particles, RNA replicons and layered DNA plasmid vectors. In this context, measles (MV), rhabdoviruses, flaviviruses and alphaviruses expressing surface antigens from viruses and other infectious agents have been subjected to immunization studies in animal models. Moreover, similar studies have been conducted with tumor antigens. It seems that MV-, rabies virus (RABV)-, vesicular stomatitis virus (VSV)-, Kunjin virus-, Semliki Forest virus (SFV)-, Sindbis virus (SIN)- and Venezuelan equine encephalitis virus (VEE)-based delivery efficiently elicits humoral and cellular immune responses in immunized animals. Furthermore, numerous cases have demonstrated protection against challenges with lethal viruses/infectious agents or with tumor cells. Most of the studies have been conducted with replication-deficient recombinant particles. However, promising results have also been obtained with layered DNA plasmid vectors. A limited number of studies have applied administration of RNA replicons, but the results have been quite encouraging. The obvious advantage to using nucleic acid-based delivery is the elimination of any risk of virus progeny production through recombination events. On the other hand, superior delivery and prolonged duration of expression can be achieved with recombinant viral particles, especially applying replication-proficient oncolytic viruses. For this reason, it is difficult to make any recommendations related to which delivery format to use, and the choice of target will play an important role in decision making.

**Figure 5:**
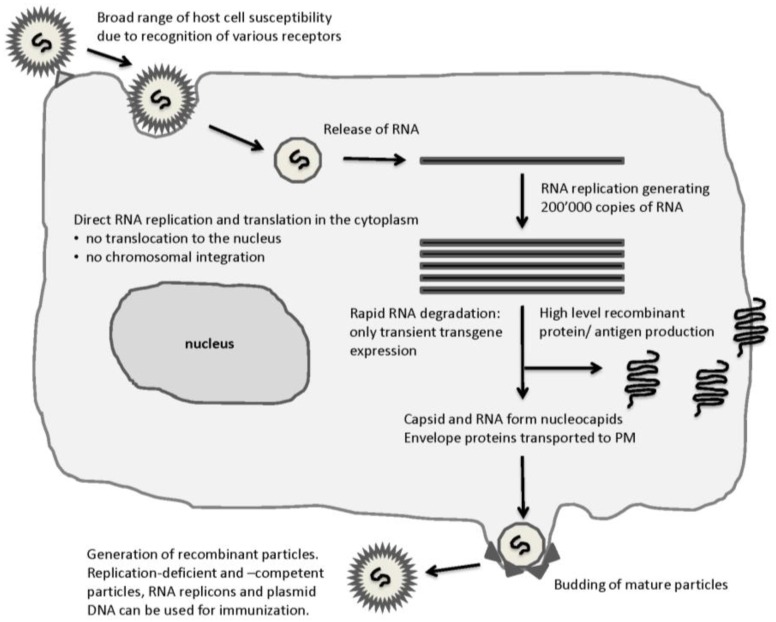
Schematic Presentation of the Life-Cycle of Self-Replicating RNA Viruses and Their Advantages. Several cell receptors are recognized providing a broad range of susceptible host cells. RNA released in the cytoplasm is immediately subjected to RNA replication and translation. Extreme RNA replication is the basis for highly efficient transgene expression.

Similarly, it is practically impossible to favor one viral vector system over another. Reverse genetics systems engineered for MV and rhabdoviruses and packaging cell lines for flaviviruses surely facilitate recombinant particle production and ease of use. Although packaging cell lines have also been generated for alphaviruses, the straightforward in vitro RNA transcription has provided the means for sufficient preparation of replicon RNA and particles for immunization studies. Obviously, plasmid DNA can be directly applied for vaccinations. In comparison to other viral vectors and also non-viral delivery systems, self-replicating RNA viruses can surely be considered competitive (Figure 5 and Table 4). An extensive comparison to other delivery systems is not within the scope of this review, so only a few examples are addressed. Clearly, adenovirus-based vaccine development and gene therapy has a longer history, which has generated a multitude [130] of vector improvements and also resulted a number of clinical trials [131,132]. Similarly, herpes simplex virus (HSV) vectors have been frequently applied and HSV-GM-CSF have, for instance, been subjected to phase I−III human clinical trials in glioblastoma and melanoma patients [133]. HSV vectors were recently approved by the FDA for use in standard patient care [134]. Related to non-viral vectors, recently dendrimer-RNA nanoparticles have demonstrated protective immunity against lethal challenges with Ebola virus, influenza H1N1 virus and *Toxoplasma gondii* after a single injection in BALB/c mice [135].

Overall, self-replicating RNA viral vectors possess several attractive features. The presence of RNA replicons provides the efficient means for rapid generation of a large number of RNA copies for immediate protein translation in the cytoplasm of host cells. Moreover, the strong subgenomic promoter utilized by alphaviruses generates extreme levels of heterologous gene expression. The transient nature of expression is also an advantage for immunization studies. Furthermore, there is no risk of integration of viral genes in the host genome as the viral RNA is degraded within 3–5 days. In the case of immunization with layered alphavirus DNA vectors, approximately 100- to 1000-fold lower doses are required compared to immunizations with conventional plasmid DNA [136].

Although strong immune responses have been obtained and protection against challenges with lethal pathogens and tumor cells have been achieved and even tumor regression observed in animals with established tumors, some further technology development is necessary. Much development has been invested in vector design including mutant vectors, enhancement signals, targeting DCs and fusion constructs. Furthermore, quite an effort has been paid to the evaluation of different target antigens and immunogens. Several studies, particularly clinical trials, have indicated that although target-specific immune responses have been obtained, further investment is required in finding the right dose for the achievement of optimal response. One area which recently has received much attention is combination therapy. Tumor-associated antigens (TAAs) have been combined with cytokines and antibodies, as well as drugs and radiation co-administered with cytokines. Additionally, optimization of adjuvant composition and stability issues in case of RNA delivery needs to be addressed. Further research in these areas will certainly provide progress and should make immunotherapy an important approach in both prophylactic and therapeutic applications.

## Figures and Tables

**Table 1:** Self-replicating RNA viral vector-based immunizations against viral diseases.

Virus	Target	Vector	Immunization	Response	Reference
Influenza	NP	SFV VLPs	mouse	systemic NP immune response	[35]
	HA	VEE VLPs	chicken	protection against influenza virus	[36]
	HA	VEE VLPs	swine	protection against influenza virus	[37]
	HA	VEE VLPs	swine	protection against influenza virus	[38]
	HA	rMV	mouse	neutralizing Abs	[39]
	cHA	VSV	mouse	protection against influenza virus	[40]
HIV	Gag	Kunjin VLPs	mouse	protection against HIV	[41]
	Env	SFV VLPs	mouse	neutralizing Abs, humoral response	[42]
	gp41	SFV-VLPs	mouse	generation of mAbs	[43]
	Env	SFV DNA	mouse	T cell and IgG immune responses	[44]
SIV	Gag-Pol	Kunjin VLPs	macaques	protection against SIV	[45]
	Env	VSV VLPs	macaques	neutralizing Abs	[46]
	Gag-Env	VSV VLPs	macaques	protection against SIV	[47]
	Gag-Env	RABV VLPs	macaques	protection against SIV	[48]
Ebola	GP	Kunjin VLPs	guinea pig	protection against Ebola	[49]
	GP	Kunjin VLPs	primate	protection against Ebola	[50]
	GP	VSV VLPs	macaques	protection against Ebola	[51,52]
	GP, NP	VEE VLPs	mouse	protection against Ebola	[53]
	NP	VEE VLPs	mouse	protection against Ebola	[54]
Lassa	G	VSV VLPs	guinea pig	protection against Lassa	[55]
	G	VEE VLPs	guinea pig	protection against Lassa	[56]
SARS-CoV	G	VEE VLPs	mouse	protection against SARS-CoV	[57]
MERS-CoV	G	MV	mouse	protection against SARS-CoV	[58]
RSV	F	MV	rat	protection against RSV	[39]
	F	VEE LNPs	mouse	protection against RSV	[59]
	F	VEE VLPs	primate	protection against RSV	[60]
MPV	F	VEE VLPs	primate	protection against MPV	[60]
Dengue	DV2-HBsAg	MV	mouse	neutralizing Abs	[61]
	DV2	MV	mouse	protection against dengue virus	[62]
	prME-E85	VEE VLPs	macaques	protection against dengue virus	[63]
	prME-E85	VEE VLPs	mouse	protection against dengue virus	[64]
HBV	MHB	SFV-VSV G	mouse	protection against HBV	[65]
	DV2-HBsAg	MV	mouse	protection against HBV	[62]
	HBsAg	MV	macaques	protection against HBV	[66]
CMV	gB-pp65/IE1	VEE VLPs	human	neutralizing Abs	[67]

Abs, antibodies; cHA, chimeric hemagglutinin; CMV, cytomegalovirus; DV2, dengue virus 2; G, glycoprotein; HA, hemagglutinin; HBV, hepatitis B virus; HBsAg, HBV surface antigen; LNPs, lipid nanoparticles; mAbs, monoclonal antibodies; MERS-CoV, Middle East respiratory syndrome coronavirus; MV, measles virus; MPV, metapneumonia virus; NP, nucleoprotein; RABV, rabies virus; RSV, respiratory syncytial virus; SARS-CoV, severe acute respiratory syndrome coronavirus; SFV, Semliki Forest virus; VEE, Venezuelan equine encephalitis virus; VLPs, virus-like particles.

**Table 2:** Self-Replicating RNA Viral Vector-Based Immunizations against Infectious Diseases.

Agent	Target	Vector	Immunization	Response	Reference
*P. falciparum*	Ag Pf332	SFV VLPs/RNA	mouse	immunological memory	[68]
*M. tuberculosis*	Ag 85A	SIN DNA	mouse	protection against *M.* *tuberculosis*	[69]
*C. botulinum*	BoNTA-Hc	SFV DNA	mouse	Ab and lymphoproliferative response	[70]
*B. abortus*	IF3	SFV VLPs	mouse	protection against *Brucella*	[71]
*B. antracis*	PA	SIN VLPs	mouse	protection against *B. antracis*	[72]
Malaria	CS	SIN VLPs	mouse	protection against malaria	[73]
*L. monocytogenes*	OVA	VSV-GP	mouse	protection against *Listeria*	[74]
Prion	PRNP	SFV VLPs	mouse	monoclonal Abs	[75]
Staphylococcus	SEB	VEE VLPs	mouse	protection against enterotoxin	[76]

Abs, antibodies; CS, circumsporozoite protein; IF3, translation initiation factor 3; MV, measles virus; MPV, metapneumonia virus; OVA, ovalbumin; PRNP, prion protein; SEB, staphylococcus enterotoxin B; SFV, Semliki Forest virus; SIN, Sindbis virus; VEE, Venezuelan equine encephalitis virus; VLPs, virus-like particles; VSV-GP, vesicular stomatitis virus pseudotyped with lymphocytic choriomeningitis glycoprotein.

**Table 3:** Self-Replicating RNA Viral Vector-Based Immunizations against Cancers.

Cancer	Target	Vector	Response	Reference
Brain	GFP, SLAM, EGFR	MV	replication in/lysis of cancer cells	[79]
	Endostatin	SFV VLPs	tumor inhibition	[89]
	miR-124	SFV-miR-124	prolonged survival	[90]
	IL-12	SFV-IL-12	prolonged survival	[91,92,93]
Breast	CEA	MV	tumor growth delay, better survival	[83]
	Neu	SIN DNA	immune responses, tumor protection	[94]
	Neu	VEE VLPs + DCs	tumor regression by transduced DCs	[95]
	VEGFR-2	SFV VLPs	tumor inhibition	[96]
Cervical	HPV E6, 7	SFV VLPs	tumor eradication	[97,98]
	HPV E7	VEE VLPs	eradication of existing tumors	[99]
	HPV E7 Epitope	Kunjin VLPs/RNA/DNA	tumor protection in mice	[88]
Colon	GM-CSF	Kunjin VLPs	regression of tumors and metastasis	[87]
	VEGFR-2	SFV VLPs	reduced tumor and metastasis growth	[96]
	LacZ	SFV RNA	tumor protection in mice	[100]
	Lac Z	SIN VLPs	anti-tumor CD8+ T-cell immunity	[101]
	IL-12	SFV VLPs	tumor elimination	[102]
	SFV	SFV VLPs	tumor growth inhibition	[103]
	IL-18	SFV VLPs	tumor regression in mice	[104]
Liver	IL-12	SFV VLPs	anti-tumor responses in woodchucks	[105,106]
Lung	HPV E6/E7	SFV + Sun + Rad	tumor-free survival	[107]
	HPV E7-CRT	SIN VLPs	long-term anti-tumor effect	[108]
	EGFP	SFV VLPs	apoptosis, tumor regression in mice	[109]
Melanoma	GM-CSF	Kunjin VLPs	tumor regression	[87]
	VEGF-2-IL-12 + Sur + β-hCG	SFV VLPs	tumor inhibition	[110]
	TRP-2	VEE VLPs	humoral and cellular immunity	[111]
	Tyr	VEE VLPs	T-cell responses, tumor protection in mice	[112]
Ovarian	CEA, NIS	MV	superior dual therapy	[81]
	IL-12	SIN VLPs	tumor targeting, eradication	[113]
	IL-18	SFV VLPs	therapeutic anti-tumor response	[91]
	GM-CSF	SFV VLPs	tumor growth inhibition	[114]
Pancreatic	Matrix protein	VSV VLPs	killing of tumor cells in vitro and in vivo	[85]
Prostate	CEA	MV	replication in/lysis of cancer cells	[79]
	PSMA	VEE VLPs	cellular and humoral immunity in mice	[115]
	STEAP	VEE VLPs	CD8+ T-cell response, tumor growth delay	[116]
	PSCA	DNA + VEE VLPs	long-term protective immune response	[117]
Sarcoma	PSA	VEE VLPs	PSA-cell clearance, tumor growth delay	[118]
Skin	SFV	SFV VLPs	tumor growth inhibition	[103]
	P1A	SFV VLPs	strong CTL-response, tumor protection	[119]

CEA, carcinoembryonic antigen; CRT, calreticulin; CTL, cytotoxic T lymphocyte; EGFP, enhanced green fluorescent protein; EGFR, epidermal growth factor receptor; GFP, green fluorescent protein; GM-CSF, granulocyte macrophage colony-stimulating factor; HPV, human papilloma virus; MV, measles virus; NIS, sodium iodide symporter; PSMA, prostate-specific membrane antigen; PSCA, prostate stem cell antigen; RABV, rabies virus; SFV, Semliki Forest virus; SIN, Sindbis virus; SLAM, signaling lymphocytic activation molecule; STEAP, six-transmembrane epithelial antigen of the prostate; Sun, sunitab; Sur, survivin; TRP, tyrosine-related protein; Tyr, melanoma antigen tyrosinase; VEE, Venezuelan equine encephalitis virus; VEGFR, vascular endothelial growth factor receptor; VLPs, virus-like particles; VSV, vesicular stomatitis virus.

**Table 4:** Comparison of Self-replicating RNA Viral Vectors with other Viral Vectors.

Viral Vector	Genome	Capacity	Special Features
Alphavirus	ssRNA	6–8 kb	broad host range, high titer, cytoplasmic RNA, extreme transient expression, no chromosomal integration, choice of DNA, RNA replicon and particle delivery
Flavivirus	ssRNA	5 kb	broad host range, packaging system, choice of DNA, RNA replicon and particle delivery
Measles virus	ssRNA	5 kb	packaging cell line, measles virus strains for immunization, cytoplasmic RNA
Rhabdovirus	ssRNA	5 kb	reverse genetics systems, broad host range cytoplasmic RNA
Adenovirus	dsDNA	>8 kb	broad host range, packaging cell line, nuclear translocation necessary, transient expression, potential integration
AAV	ssDNA	<4 kb	multiple AAV serotypes for avoiding immune responses, nuclear translocation necessary, chromosomal integration
Herpes simplex virus	dsDNA	30–40 kb	large packaging capacity, nuclear translocation necessary, latent long-term transgene expression after integration
Lentivirus	dsRNA	8 kb	transduction of dividing and non-dividing cells, nuclear translocation necessary, chromosomal integration
Retrovirus	dsRNA	4 kb	transduction of only dividing cells, nuclear translocation necessary, chromosomal integration
Vaccinia	dsDNA	25 kb	large packaging capacity, nuclear translocation necessary

AAV, adeno-associated virus; dsDNA, double-stranded DNA; ssDNA, single-stranded DNA; ssRNA, single-stranded RNA.

## Abbreviations

The following abbreviations are used in this manuscript:

MDPI	Multidisciplinary Digital Publishing Institute
DOAJ	Directory of open access journals
CEA	Carcinoembryonic antigen
CMV	Cytomegalovirus
CRPC	Castration-resistant metastatic prostate cancer
CRT	Calreticulin
CTL	Cytotoxic T-lymphocyte
DC	Dendritic cell
EGFP	Enhanced green fluorescent protein
EGFR	Epidermal growth factor receptor
GFP	Green fluorescent protein
GM-CSF	Granylocyte macrophage colony-stimulating factor
GoI	Gene of interest
HA	Hemagglutinin
HBV	Hepatitis B virus
HCC	Hepatocellular carcinoma
HPV	zhuman papilloma virus
IF3	Initiation factor 3
IFN	Interferon
IL	Interleukin
ISG	Interferon-stimulated gene
MERS-CoV	Middle East respiratory syndrome coronavirus
MHC	Major histocompatibility complex
miRNA	Micro RNA
MLN	Mediastinal lymph nodes
MV	Measles virus
NIS	Sodium iodide symporter
NP	Nucleoprotein
PDAC	Pancreatic ductal adenocarcinoma
PSMA	Prostate-specific membrane antigen
PSCA	Prostate stem cell antigen
RABV	Rabies virus;
RSV	Respiratory syndrome virus
SARS-CoV	Severe acute respiratory syndrome coronavirus
SFV	Semliki Forest virus
SEB	Staphylococcus enterotoxin B
SIN	Sindbis virus
ssRNA	Single-stranded RNA
STEAP	Six-transmembrane epithelial antigen of the prostate
TAA	Tumor-associated antigen
TRAMP	Transgene adenocarcinoma of the mouse prostate
TRP	Tyrosine-related protein
Tyr	Tyrosinase
VEE	Venezuelan equine encephalitis virus
VEGFR	Vascular endothelial growth factor receptor
VLPs	Virus-like particles
VSV	Vesicular stomatitis virus
WHV	Woodchuck hepatitis virus
